# Self-Perceived Competencies and Attitudes on Palliative Care in Undergraduate Nursing Students: A Multicenter Descriptive Study

**DOI:** 10.3390/nursrep14030188

**Published:** 2024-09-22

**Authors:** Cinzia Lo Iacono, Emanuele Amodio, Giuseppe Vella, Maria Caruso, Giuseppe D’Anna, Angelo Gambera, Maurizio Soresi, Giuseppe Intravaia, Roberto Latina

**Affiliations:** 1 Terminal Cancer Patient Assistance Society (SAMOT Onlus), Via della Libertà 193, 90143 Palermo, Italy; cinzia.loiacono01@unipa.it (C.L.I.); giuseppe.intravaia@unipa.it (G.I.); 2 Department of Health Promotion, Maternal and Infant Care, Internal Medicine and Medical Specialties (PROMISE), University of Palermo, 90143 Palermo, Italy; emanuele.amodio@unipa.it (E.A.); maurizio.soresi@unipa.it (M.S.); roberto.latina@unipa.it (R.L.); 3 Azienda Ospedaliera Universitaria Policlinico “G Martino”, University of Messina, 98124 Messina, Italy; maria.caruso@unime.it; 4 Azienda Ospedaliera Universitaria Policlinico “P. Giaccone”, University of Palermo, 90143 Palermo, Italy; giuseppe.danna@unipa.it; 5 Azienda Ospedaliero Universitaria Policlinico “G. Rodolico—San Marco”, University of Catania, 95124 Catania, Italy; a.gambera@ao-ve.it

**Keywords:** self-assessment, competencies, attitudes, palliative care, dying, undergraduate, nursing

## Abstract

*Introduction:* Caring for the dying can generate anxiety and emotional distress, particularly in nursing students, and perceived competence could play a crucial role in enabling nurses to perform their duties with greater confidence. Unfortunately, few studies describe the relationship between students’ nursing attitudes and perceived self-efficacy in palliative care (PC). To overcome this gap, this survey aimed to assess the attitudes towards dying patients and the perceived competence of nursing students in palliative care at different universities in the south of Italy. *Methods:* A cross-sectional study was conducted from September 2022 to March 2023 involving nursing students from the three major Sicilian universities (Italy). The study included a survey investigating socio-demographic characteristics, palliative care training, knowledge about pain management, and previous experience with dying. Moreover, the Professional Competence of the Core Curriculum in Palliative Care Nursing (CCPCN) questionnaire and the Frommelt Attitudes Toward Care of the Dying—B Italian version (FATCOD-B-I) assessed competencies and emotional attitudes. *Results:* A total of 1913 nursing students were recruited, of which 71.3% were females, and 53.9% were in the age range of 18 to 21 years. In the multivariable analysis, practical PC training was a substantial factor in enhancing competencies (Adj-OR 2.78 [95% CI = 2.12–3.65]). Male students had higher competence odds (Adj-OR 1.38 [95% CI = 1.14–1.66]), and perceived knowledge strongly correlated with self-assessed competence. Advancement in academic years also positively influenced competence self-assessment (Adj-OR 1.98 [95% CI = 1.75–2.24]). Regarding emotional attitudes, a per-quartile increase in competence score was found to improve the attitude score (Adj-OR 1.24 [95% CI = 1.13–1.35]). *Conclusions:* Nursing students gain valuable experience during clinical experience. PC training and perceived knowledge of PC significantly increase nurses’ competencies, and the latter seem to be strongly associated with attitudes. Thus, introducing palliative care education into nurses’ core curricula could be a way to reduce anxiety and emotional distress in young students.

## 1. Introduction

Globally, it is estimated that 40 million individuals require palliative care (PC) annually, a demand projected to rise significantly in the forthcoming years, particularly in the realm of end-of-life care and dying [1,2]. The primary objective of PC is to enhance the quality of life for patients and their families by preventing and treating pain, along with addressing physical, psychosocial, and spiritual issues until death and beyond. PC neither hastens nor postpones death but views it as a natural process, employing an interdisciplinary approach that emphasizes personalized care delivered by various healthcare practitioners [2,3].

Among healthcare practitioners, nurses spend the most time with dying patients and their families, often witnessing the process of death [4,5]. They are a crucial support system for the patient, family, and caregivers [6,7].

Generally, PC can be an emotionally challenging and sometimes threatening experience for nurses because it needs advanced skills, appropriate attitudes, and a natural acceptance of dying and death [8,9]. Death can trigger intense emotional reactions, influenced by several factors, including individual perceptions of death, values, beliefs, past experiences, and cultural characteristics [10,11]. Dealing with death and caring for patients in palliative care can induce stress in nurses, eliciting feelings of helplessness, nervousness, guilt, regret, sadness, anxiety, frustration, anger, and repulsion, possibly because the propensity to care for dying individuals is not innate [12,13].

Caring for the dying can generate anxiety, terror, and emotional distress, particularly in nursing students due to fear of their reactions to death, loss of control, and perceived inability to provide adequate and sensitive support to patients and family members [14]. Such feelings can foster negative attitudes towards the care of dying patients [15].

Positive attitudes, specific knowledge, and perceived competence (self-efficacy) in PC and end-of-life care are pivotal to adequately supporting these patients and influencing the quality of palliative care [9,16]. According to the Shared Theory of Palliative Care, the ability of nurses to provide competent palliative care is linked to their perceived competence [17]. Perceived competence is crucial in enabling nurses to confidently perform their duties [9].

The literature suggests that undergraduate nursing students lack the competence to provide palliative care and feel unprepared for palliative care and encountering death [18,19,20]. People tend to avoid situations requiring a skill when perceived competence is low. Conversely, individuals demonstrate more persistent determination toward completing an action when perceived competence is high. Therefore, educators should evaluate students’ perceived competence as it can impact the provision of palliative care [21]. 

Studies worldwide on nursing students’ attitudes towards care of the dying have identified several factors that can positively or negatively influence these attitudes, showing often contradictory results. Researchers have widely investigated death attitude as a possible factor impacting attitudes toward care of the dying [22]. The literature suggests that nurses and nursing students who have a more positive attitude towards death are more likely to have a positive attitude towards end-of-life care [4,11,23,24,25,26,27,28].

The training received in palliative and end-of-life care is the factor that can most influence attitudes towards care of the dying [29,30]. Studies conducted in different parts of the world have shown that nursing students’ attitudes improve with specific education in palliative care [30,31,32,33], although in a study conducted in Indonesia, training had no relation to students’ attitudes to care of the dying [34]. Being a woman [11], year of study, and older age are significant predictors of positive attitudes [9,26], while religiosity and cultural and social circumstances could also be influential [23,35]. Religiosity, in fact, in a study carried out on a Swedish–Iranian sample, increased negative attitudes towards caring for people who were dying [24].

A multicenter European study involving Spain, England, and Italy assessed nursing students’ attitudes towards caring for dying patients using the FATCOD-B scale. The study found that students across all three countries showed moderate attitudes, with scores falling in the middle range of the scale. Italian students demonstrated slightly more positive attitudes compared to their Spanish and English counterparts, although this difference was not statistically significant. Interestingly, the study reported that various factors such as age, sex, year of study, religious beliefs, palliative care nursing education, previous experience with dying patient care, and personal bereavement did not significantly influence these attitudes. This suggests a relatively consistent baseline attitude among nursing students across these countries, regardless of personal or educational factors [36]. An Italian study showed that higher scores were significantly associated with training in palliative care and experience with terminally ill patients. Moreover, students described more negative attitudes when they perceived patients losing hope of recovering and patients’ family members interfering with health professionals’ work. They believe caring for a terminally ill patient is a helpful learning experience but feel they need to be adequately prepared in practice [37]. Specific training should improve nursing students’ knowledge and self-perceived competence about palliative care and end-of-life care, thus enhancing a positive death attitude to better deal with dying patients, increasing self-awareness on death, and allowing to provide quality care [35,38]. However, to orientate nursing education appropriately, more knowledge about nursing students’ self-perceived competence in palliative care and attitudes towards care of the dying is needed [37,39].

Only a few studies have described the relationship between nursing students’ attitudes and perceived self-efficacy in palliative care [9,40], particularly on undergraduate nursing students.

In order to obtain a clearer picture of the attitudes towards dying patients and the perceived competence of nursing students in palliative care, the objective of this survey is to assess these two essential aspects in different universities in the south of Italy.

## 2. Methods

### 2.1. Design, Instruments, and Data Collection

This cross-sectional study involved 1913 nursing students from three Italian universities: the University of Messina, the University of Palermo, and the University of Catania. The study utilized a two-section questionnaire. These participants represented 89% of the total enrolled nursing students across the three universities (N = 2150). Students were contacted using the official university email databases for nursing degree programs, ensuring a comprehensive reach to the target population. Non-responders were proportionally distributed among the three universities: 97 from Messina (10% of the total students), 85 from Palermo (11%), and 30 from Catania (7%). The first questionnaire section collected data on socio-demographic characteristics such as sex, age, year of study, PC education, perceived degree of knowledge about pain management, previous experience with dying individuals, previous bereavement experience, current bereavement experience, religion, and university affiliation. In the second section, two specific instruments were used:

1. Professional Competence of the Core Curriculum in Palliative Care Nursing (Professional Competence-CCPCN) [41]: This instrument, composed of 24 items, was used to measure students’ perceived competence in palliative care. Each item is scored on a scale from 0 to 10, with higher scores indicating higher perceived competence. The total score was divided into quartiles (q1, q2, q3, q4).
2. Frommelt Attitudes Toward Care of the Dying—B Italian version (FATCOD-B-I) [42]: This questionnaire, consisting of 30 items, gauges attitudes towards care of the dying. The scoring is based on a five-point Likert scale ranging from “strongly agree” to “strongly disagree”. Fifteen items (1, 2, 4, 10, 12, 16, 18, 20, 21, 22, 23, 24, 25, 27, and 30) are formulated positively, and the remaining fifteen negatively. The response scale is a 5-point Likert-type scale. For positive items, the score ranges from 1 (“Strongly disagree”) to 5 (“Strongly agree”). For negative items, the score is reversed. The total score ranges between a minimum of 30 and a maximum of 150, with higher scores indicating more positive attitudes. Scores > 65% of the total possible score (>97.5) were considered as positive attitudes, those between 50% and 65% (>75–<97.5) as neutral, and those below 50% (<75) as negative. The total score was categorized into equal quartiles (eq1, eq2, eq3, eq4) to assess emotional attitudes. Data were collected over the period from September 2022 to March 2023. The questionnaire was delivered to participants online through a survey platform, ensuring convenience and anonymity.

### 2.2. Inclusion and Exclusion Criteria

All students, regardless of sex and with no age limit, were included in the 1st, 2nd, and 3rd years, as well as out-of-course students of the Degree Courses in Nursing at each of the three universities. This includes both native speakers and international students with an excellent command of the Italian language. International students unfamiliar with the Italian language, or those who did not consent to participate, were excluded.

### 2.3. Bachelor’s Degree in Nursing Science and Palliative Care Curricula

In Italy, a bachelor’s degree in nursing science spans three years, amounting to 180 European Credit Transfer System (ECTS) credits (1 ECTS = 30 h), with at least 70 ECTS credits dedicated to internships to develop specific skills in various clinical areas. Training in palliative care in the three universities under study is organized as reported below:

(a) The Palermo program, in the third year, provides 3 ECTS credits of a compulsory theoretical module;
(b) The Messina program offers 2 ECTS credits in the second year;
(c) The Catania program does not have a compulsory dedicated training but allows students to participate in an optional didactic activity.

Clinical placements for palliative care apprenticeships are provided in all three universities, but they are limited and not compulsory.

### 2.4. Statistical Analysis

Quantitative variables are summarized using the mean and standard deviation (or median and inter-quartile range where appropriate). Qualitative variables are expressed as absolute frequencies and percentages.

In the univariate analysis, Chi-square tests were performed to examine the association between competence or emotional attitude quartiles (based on Professional Competence-CCPCN and FATCOD-B-I scores, respectively) and categorical variables from the first part of the questionnaire. Age was stratified into categories reflecting presumed variations in education level. Quartiles for each scale were derived from the score distributions, arranging the dataset in ascending order. Thresholds for quartiles for each scale were established according to the distribution, segmenting the dataset into four groups of equal size. Each participant was subsequently allocated into a specific quartile based on their total score. For instance, a participant with a score falling within the range of the first quartile for the Professional Competence-CCPCN scale was assigned to the first competence quartile (q1), and the process was analogous for the FATCOD-B-I scale. Quartile analysis is a robust method for examining the distribution of scores and allows for comparing group characteristics across the spectrum of the dataset. Subsequently, we performed multinomial ordinal regression to define variables able to predict competence and emotional attitudes quartiles (based on Professional Competence-CCPCN and FATCOD-B-I scores, respectively). The adjusted odds ratios (Adj-OR) and their 95% confidence intervals (CI) were then calculated.

A *p*-value of less than 0.05 was considered statistically significant. R Studio software version 2023.12.1 was used for the statistical analyses, including the following packages: dplyr, ggplot2, car, and nnet for ordinal multinomial regression.

### 2.5. Ethical Issues

The study was conducted following the principles of the Helsinki Declaration and was approved by the Local Ethics Committee (Ethics Committee Palermo 1 N. 05/2022, 11 May 2022). Students received detailed oral and written information about the study. Those who agreed to participate provided informed consent. The anonymity of the participants was guaranteed throughout the entire data analysis and reporting process.

## 3. Results

A total of 1913 students were included in the analyses (Table 1), a robust proxy of the total population of nursing students in Sicily (89%). Distribution by university was as follows: University of Messina (42.6%), University of Palermo (35.5%), and University of Catania (21.8%). The cohort predominantly consisted of females (71.3%) aged 18 to 21 years (53.9%). The competence score quartiles, based on even distribution among the participants, are detailed in Table 2. Univariate analyses showed a significant association between emotional attitudes and competence, particularly notable in the upper quartiles. For instance, a higher proportion of students in the top emotional attitude quartile (eq4) were also in the highest competence quartile (q4, 37%), with the converse true for the lowest quartile (q1, 22%) (*p* < 0.001). A significant variation in distribution among the universities was observed across competence quartiles (*p* < 0.001), with the University of Messina contributing more to the highest quartile and the University of Catania contributing more to the lowest. Sex showed no significant difference across quartiles, whereas age was statistically significantly associated with different competency quartiles (*p* < 0.001). The academic year of study was a differentiator, with first-year students primarily in the lowest quartile (50%) and those in their third year or higher in the top quartiles (q3 and q4) (*p* < 0.001). Palliative care (PC) training also correlated significantly with higher competence quartiles, where students who completed compulsory theoretical training were predominantly in the upper half (q3 and q4).

Knowledge about pain management was significantly associated with competence level, with students self-rating as “excellent” being mostly in the top quartile (*p* < 0.001). Experience with dying individuals was associated with higher competence, but bereavement experience and religious beliefs were not.

Students were also categorized into quartiles for emotional attitude based on FATCOD-B-I scores, with significant differences in distribution among universities (*p* = 0.0035) (Table 3), and the study year was also significantly related to higher emotional attitude quartiles (*p* = 0.002). The University of Messina contributed more to the highest quartile, while the University of Catania contributed more to the lowest. For emotional attitudes, there were also significant differences in distribution among universities (*p* = 0.0035).

The level of PC education showed a trend where those with compulsory theoretical training were more likely to be in the upper quartile, whereas those without it were in the lower quartile (*p* = 0.013); thus, the differences in curricula between universities with compulsory training (Messina and Palermo) and those without (Catania) could explain such differences.

**Figure 1:**
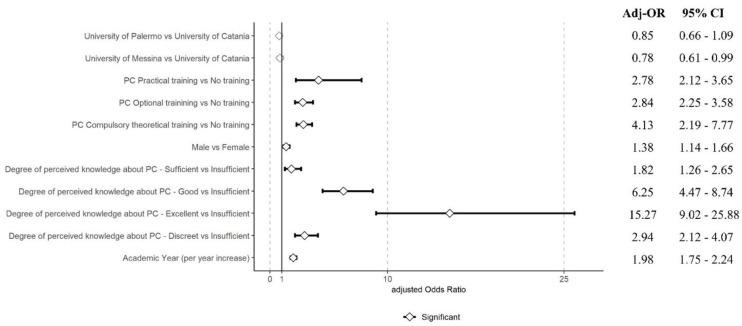
Adjusted odds ratios for Professional Competence-CCPCN scores.

As shown in Figure 1, multinomial ordinal regression revealed that practical PC training substantially enhanced competencies (Adj-OR 2.78 [95% CI = 2.12–3.65]). Male students had higher competence odds (Adj-OR 1.38 [95% CI = 1.14–1.66]), and perceived knowledge strongly correlated with self-assessed competence. Advancement in academic years also positively influenced competence self-assessment (Adj-OR 1.98 [95% CI = 1.75–2.24]).

**Figure 2:**
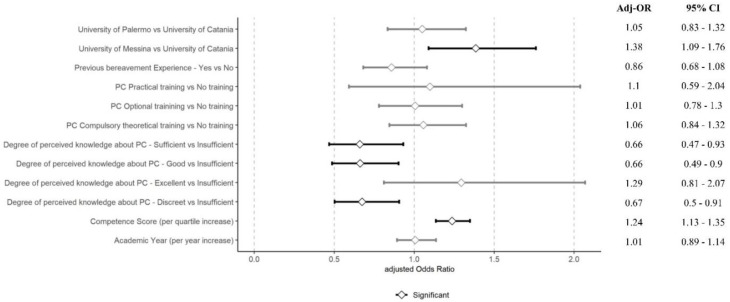
Adjusted odds ratios for FATCOD-B-I scores.

Regarding emotional attitudes, knowledge of PC showed mixed effects on attitude, with excellent knowledge not significantly affecting attitude scores. However, a per-quartile increase in competence score improved the attitude (Adj-OR 1.24 [95% CI = 1.13–1.35]), although the academic year did not significantly impact it. Figure 2 illustrates the adjusted odds ratios for the FATCOD-B-I score, visually representing the relationships between various factors and emotional attitudes towards care of the dying. The figure highlights the significant positive association between competence scores and emotional attitudes, while also showing the relatively minimal impact of other factors such as academic year and PC education on these attitudes.

## 4. Discussion

This study explored the perceived competence in palliative care and attitudes towards dying patient care in Italian nursing students using the Professional Competence-CCPCN and the FATCOD-B-I, respectively. Overall, our findings revealed a spectrum of perceived competence ranging from low to intermediate, aligning with Zhou et al. [22] and Max and Mackenzie [40], who reported similar levels of self-efficacy in palliative care among nursing students in China and the United States, respectively. Progression in the academic curriculum proved to be a pivotal factor; in fact, perceived self-competence in palliative care was observed to improve from the first to the third year in our study. Regarding attitudes towards caring for dying patients, our results showed that nursing students scored positively, although slightly less so than in another Italian study [36,37]. However, Italian results are only sometimes in line with those from other countries. In China [22], Sweden [30], Switzerland [9], and the United States [43], the results are better than the Italian ones. However, the same cannot be said of those in Mongolia [44], England [31], Iran [32], and India [45]. The wide variability in results between countries could be due to cultural, social, and religious differences, which may influence students’ attitudes towards death and dying patients [35].

Moreover, our results show that more than 70% of the sample stated that they optimally perceive pain management in terms of knowledge and attitudes. This result is probably overestimated, as some studies observed that Italian education pays little attention to palliative care training and presents limited knowledge and attitudes toward pain management [36,46], particularly about the combination of interventions [47]. Moreover, even nurses report limited attitudes and knowledge about acute and chronic pain assessment and treatment, and not only in Italy [48,49]. 

The link between the level of PC education and higher competence quartiles indicates the essential role of mandatory theoretical training. This requirement needs to be uniformly implemented across Italian universities. Although the Italian Ministries of Research and Universities strongly encourage palliative care training of healthcare workers, to date, theoretical and clinical curricula on PC are compulsory only in two out of the three surveyed universities, and this problem is present at a national and European level [47,50]. In 14 European countries (56%), PC was not identified as mandatory in undergraduate nursing education. The broad awareness and use of the EAPC 2004 Guide show how policy measures can influence the development of palliative care education. Recommendations have been built and focus on fostering the use of this guide and implementing policy measures to ensure that palliative care nursing is recognized and certified as a specialty in all European countries [51]. Moreover, a recent scoping review showed that using multiple learning methods increases undergraduate student nurses’ knowledge and positive attitudes towards providing palliative care and clinical exposure to meaningful learning opportunities with patients experiencing severe life-threatening illnesses, which facilitates learning and enables a change of attitudes. Furthermore, from an educational perspective, there needs to be more integration of palliative care philosophy and conceptual frameworks. Issues such as crowded curricula, a lack of nurse educators, and a lack of expertise in teaching palliative care within nursing faculties, as well as issues related to the timing and delivery of palliative care education, must also be addressed. 

Although our results demonstrated the importance of enhancing competencies through practical training in palliative care, clinical placements for nursing students pose significant barriers to increased learning opportunities in palliative and end-of-life care. Although progress has been made towards integrating PC curricula into undergraduate nurse education in Italy, this research shows that further curriculum development is needed to build the workforce capacity to meet the increasing demand for primary palliative care. Adding content to an overcrowded nursing curriculum is challenging, as is also the case for other health professionals such as doctors. However, it is not impossible. Several researchers and educators recommend an integrated, spiral approach with multidisciplinary teaching that introduces foundational concepts early to prepare students for clinical encounters with people who require palliative care [52]. Further, from a clinical practice perspective in palliative care, there are difficulties in providing clinical placement [53,54]. Moreover, health policy, clinical, and academic nursing leaders should prioritize the integration of palliative care content into the curricula across nursing schools in the face of increasing palliative and end-of-life care needs in patients. Nursing schools should ensure that students are adequately prepared by designing culturally and socioeconomically relevant curricula, integrating theoretical and experiential learning and offering students a thorough understanding of palliative and end-of-life care. Clinical staff and nursing instructors should support students emotionally and guide them in patient care [55,56].

Notably, students with PC education displayed better attitudes toward the care of the dying. However, prior experience with dying individuals and bereavement experiences did not exhibit relations to any significant difference across quartiles. A recent meta-analysis of qualitative studies describing nursing students’ experiences when caring for dying patients and their families found that nursing students advocated for more caring for patients’ families [55].

Our results show that males had better attitudes compared to female students about care of the dying. These results, however, contrast with other studies in the literature, where females showed better attitudes than males in the management of dying patients [56].

Lastly, while the simultaneous per-year increase in academic experience and perceived self-efficacy suggests that experience and progression in studies positively influence self-perceived competencies in PC, on the contrary, the academic year was not significant for attitudes. In the literature, those with the most clinical experience had more positive attitudes toward caring for the dying, and senior students were found to have positive attitudes toward caring for the dying. These results are supported by Petrolongo and Tooteaker [43], who found a significant positive relationship between years of experience and caring for dying patients. The more academic experience and exposure to death one has, the higher their awareness and ability to manage and adapt are.

### Limitations

Despite the large sample of surveyed students (89% of the total nursing students in the whole region) and the significance of the results, our study could have some limitations that should be considered. In particular, using a convenience sample, although representative of one region’s nursing student population, limits the generalizability of our findings. Another notable limitation of this study is its reliance on self-reported measures of competence and attitudes. While the consistency observed across our detailed, multi-item questionnaire suggests coherent self-perception among participants, we acknowledge that self-perceived competencies may not always accurately reflect actual knowledge or skills. Future research could address these limitations by incorporating objective measures of palliative care knowledge and skills alongside self-reported data and extending the analysis beyond regional boundaries to better understand nursing students’ competencies and attitudes on palliative care on a broader scale.

## 5. Conclusions

This study revealed low to intermediate levels of perceived palliative care competence among Italian nursing students, which improved with academic progression. Practical training was significantly linked to enhanced competencies and attitudes towards end-of-life care. These findings underscore the need to integrate comprehensive palliative care education, including practical experiences, earlier in nursing curricula. Such integration could address competency gaps and improve attitudes towards end-of-life care. Future research should examine the long-term impacts of enhanced palliative care education on nursing practice and patient outcomes. Developing competencies and positive attitudes in palliative care is crucial for preparing nursing students to provide high-quality end-of-life care, emphasizing the importance of targeted educational interventions and clinical experiences in nursing education.

## Figures and Tables

**Table 1:** Demographic and experiential characteristics of the nursing students.

Students’ Characteristics	University of Catania N = 417	University of Messina N = 816	University of Palermo N = 680
	n (%)	n (%)	n (%)
**Sex**			
Female	299 (71.7)	609 (74.6)	457 (67.2)
Male	118 (28.3)	207 (25.4)	223 (32.8)
**Age (years)**			
18–21	202 (48.4)	431 (52.8)	398 (58.5)
22–25	145 (34.8)	248 (30.4)	173 (25.4)
26–30	49 (11.8)	89 (10.9)	57 (8.4)
>30	21 (5.0)	48 (5.9)	52 (7.6)
**Year of Study**			
First	153 (36.7)	365 (44.7)	258 (37.9)
Second	134 (32.1)	248 (30.4)	254 (37.4)
Third or +	130 (31.2)	203 (24.9)	168 (24.7)
**PC Education**			
Compulsory theoretical training	81 (19.42)	429 (52.6)	250 (36.8)
Optional training	16 (3.8)	160 (19.6)	116 (17.1)
Practical training	10 (2.4)	14 (1.7)	11 (1.6)
No	310 (74.3)	213 (26.1)	303 (44.6)
**Perceived degree of knowledge about pain management**			
Insufficient	27 (6.5)	73 (9)	102 (15)
Good	156 (37.4)	309 (37.9)	195 (28.7)
Moderate	170 (6.5)	282 (34.6)	265 (39)
Sufficient	49 (11.8)	96 (11.8)	93 (13.7)
Excellent	15 (3.6)	56 (6.9)	25 (3.7)
**Previous experience with dying individuals**			
Internship in PC	15 (3.6)	38 (4.7)	16 (2.4)
Internship in other settings	143 (34.3)	90 (11.0)	170 (25)
Personal experience	76 (18.32)	235 (28.8)	158 (23.2)
Working experience	10 (2.4)	20 (2.5)	17 (2.5)
No	173 (41.5)	433 (53.1)	319 (46.9)
**Previous bereavement experience**			
Yes	348 (83.9)	701 (85.9)	568 (83.5)
No	69 (16.5)	115 (14.1)	112 (16.5)
**Current bereavement experience**			
No	350 (83.9)	685 (83.9)	576 (84.7)
I am preparing for the death of a loved one	14 (3.4)	37 (4.5)	24 (3.5)
I am grieving for a loved one who recently passed away	53 (12.7)	94 (11.5)	80 (11.8)
**Religion**			
Agnostic or atheist	114 (27.3)	143 (17.5)	199 (29.3)
Catholic Christians	286 (68.6)	646 (79.2)	458 (67.4)
Other (Muslim, Jewish, Protestant)	17 (4.1)	27 (3.3)	23 (3.4)

**Table 2:** Competence quartiles from the Professional Competence-CCPCN questionnaire scores.

Competence Score Quartile									
Variables	q1	%	q2	%	q3	%	q4	%	*p*-Value
**N**	480		477		480		476		
**Emotional attitudes (%)**									<0.001
eq1	149	29%	146	28%	134	26%	85	17%	
eq2	110	24%	123	27%	127	27%	103	22%	
eq3	127	25%	122	24%	128	25%	126	25%	
eq4	94	22%	86	20%	91	21%	162	37%	
**University (%)**									<0.001
Catania	84	20%	145	35%	113	27%	75	18%	
Messina	203	25%	167	20%	192	24%	254	31%	
Palermo	193	28%	165	24%	175	26%	147	22%	
**Sex (%)**									0.13
Female	360	26%	344	25%	331	24%	330	24%	
Male	120	22%	133	24%	149	27%	146	27%	
**Age (years) (%)**									<0.001
18–21	308	30%	253	25%	225	22%	245	24%	
22–25	87	15%	156	28%	180	32%	143	25%	
26–30	43	22%	47	24%	46	24%	59	30%	
30+	42	35%	21	17%	29	24%	29	24%	
**Academic year (%)**									<0.001
First	388	50%	148	19%	99	13%	141	18%	
Second	71	11%	215	34%	184	29%	166	26%	
Third or +	21	4%	114	23%	197	39%	169	34%	
**PC education (%)**									<0.001
Compulsory theoretical training	65	9%	176	23%	255	34%	264	35%	
No	355	43%	218	26%	139	17%	114	14%	
Optional training	58	20%	78	27%	73	25%	83	28%	
Practical training	2	6%	5	14%	13	37%	15	43%	
**Perceived degree of knowledge about pain management (%)**									<0.001
Insufficient	125	62%	47	23%	17	8%	13	6%	
Sufficient	84	35%	78	33%	49	21%	27	11%	
Moderate	158	22%	211	29%	215	30%	133	19%	
Good	95	14%	133	20%	185	28%	247	37%	
Excellent	18	19%	8	8%	14	15%	56	58%	
**Previous experience with dying individuals (%)**									<0.001
Internship in another setting	22	5%	108	27%	156	39%	117	29%	
Internship in PC	1	1%	16	23%	24	35%	28	41%	
No	318	34%	227	25%	182	20%	198	21%	
Personal experience	136	29%	116	25%	103	22%	114	24%	
Working experience	3	6%	10	21%	15	32%	19	40%	
**Previous bereavement experience (%)**									0.96
No	71	24%	75	25%	77	26%	73	25%	
Yes	409	25%	402	25%	403	25%	403	25%	
**Current bereavement experience (%)**									0.78
I am grieving for a loved one who recently passed away	64	28%	50	22%	54	24%	59	26%	
I am preparing for the death of a loved one	16	21%	22	29%	20	27%	17	23%	
No	400	25%	405	25%	406	25%	400	25%	
**Religion (%)**									0.08
Agnostic or atheist	117	25%	118	26%	135	29%	92	20%	
Catholic Christian	349	25%	340	25%	330	24%	367	26%	
Other (Muslim, Jewish, Protestant)	14	22%	19	29%	15	23%	17	26%	

**Table 3:** Emotional attitudes quartiles from the FATCOD-B-I questionnaire scores.

Emotional Attitudes Quartiles									
Variables	eq1	%	eq2	%	eq3	%	eq4	%	*p*-Value
**N**	514		463		503		433		
**Competence score quartile (%)**									<0.001
q1	149	31%	110	23%	127	26%	94	20%	
q2	146	31%	123	26%	122	26%	86	18%	
q3	134	28%	127	26%	128	27%	91	19%	
q4	85	18%	103	22%	126	26%	162	34%	
**University (%)**									0.0035
Catania	129	31%	104	25%	105	25%	79	19%	
Messina	193	24%	184	23%	220	27%	219	27%	
Palermo	192	28%	175	26%	178	26%	135	20%	
**Sex (%)**									0.20
Female	358	26%	343	25%	367	27%	297	22%	
Male	156	28%	120	22%	136	25%	136	25%	
**Age (years) (%)**									0.57
18–21	290	28%	243	24%	268	26%	230	22%	
22–25	152	27%	147	26%	148	26%	119	21%	
26–30	42	22%	47	24%	52	27%	54	28%	
30+	30	25%	26	21%	35	29%	30	25%	
**Academic year (%)**									0.002
First	234	30%	167	22%	183	24%	192	25%	
Second	169	27%	171	27%	174	27%	122	19%	
Third or +	111	22%	125	25%	146	29%	119	24%	
**PC education (%)**									0.013
Compulsory theoretical training	173	23%	187	25%	218	29%	182	24%	
No	261	32%	195	24%	189	23%	181	22%	
Optional training	72	25%	74	25%	84	29%	62	21%	
Practical training	8	23%	7	20%	12	34%	8	23%	
**Perceived degree of knowledge about pain management (%)**									0.0025
Insufficient	55	27%	41	20%	53	26%	53	26%	
Sufficient	75	32%	56	24%	61	26%	46	19%	
Moderate	192	27%	193	27%	187	26%	145	20%	
Good	174	26%	157	24%	180	27%	149	23%	
Excellent	18	19%	16	17%	22	23%	40	42%	
**Previous experience with dying individuals (%)**									0.505
Internship in another setting	109	27%	106	26%	106	26%	82	20%	
Internship in PC	13	19%	17	25%	24	35%	15	22%	
No	252	27%	217	23%	242	26%	214	23%	
Personal experience	129	28%	112	24%	123	26%	105	22%	
Working experience	11	23%	11	23%	8	17%	17	36%	
**Previous bereavement experience (%)**									0.016
No	85	29%	54	18%	74	25%	83	28%	
Yes	429	27%	409	25%	429	27%	350	22%	
**Current bereavement experience (%)**									0.805
I am grieving for a loved one who recently passed away	61	27%	59	26%	51	22%	56	25%	
I am preparing for the death of a loved one	22	29%	18	24%	17	23%	18	24%	
No	431	27%	386	24%	435	27%	359	22%	
**Religion (%)**									0.428
Agnostic or atheist	115	25%	127	27%	114	25%	106	23%	
Catholic Christian	385	28%	321	23%	371	27%	309	22%	
Other	14	22%	15	23%	18	28%	18	28%	

## Data Availability

Data are contained within the article. Clinical Resources: Palliative Care in Acute & Critical Care Settings—American Association of Critical-Care Nurses: https://www.aacn.org/clinical-resources/palliative-end-of-life (accessed on 1 April 2024); Palliative Care and the Nurse’s Role—University of UTAH: https://sigma.nursingrepository.org/server/api/core/bitstreams/2ef4fee9-2c6c-4fad-ae38-6b33aa6df5eb/content (accessed on 1 April 2024); CARES: Competencies And Recommendations for Educating Undergraduate Nursing Students Preparing Nurses to Care for the Seriously Ill and their Families—American Association of Colleges of Nursing: https://www.aacnnursing.org/Portals/0/PDFs/Teaching-Resources/New-Palliative-Care-Competencies.pdf (accessed on 1 April 2024)

## Public Involvement Statement

There was no public involvement in any aspect of this research.

## Guidelines and Standards Statement

This manuscript was drafted against the STROBE guidelines for cross-sectional studies. This study underscores the importance of integrating practical palliative care training early in nursing education to enhance clinical competence and foster positive attitudes towards caring for dying patients, ultimately improving the quality of end-of-life care.
